# Cilostazol Renoprotective Effect: Modulation of PPAR-γ, NGAL, KIM-1 and IL-18 Underlies Its Novel Effect in a Model of Ischemia-Reperfusion

**DOI:** 10.1371/journal.pone.0095313

**Published:** 2014-05-09

**Authors:** Diaa Ragab, Dalaal M. Abdallah, Hanan S. El-Abhar

**Affiliations:** 1 Department of Pharmacology and Toxicology, October Six University, Giza, Egypt; 2 Department of Pharmacology and Toxicology, Cairo University, Cairo, Egypt; National Centre for Scientific Research “Demokritos”, Greece

## Abstract

Cilostazol, a phosphodiesterase-III inhibitor, reportedly exhibits positive effects against ischemia/reperfusion (I/R)-induced injury in several models. However, its potential role against the renal I/R insult has not been elucidated. To test whether the PPAR-γ (of peroxisome proliferator activated receptor gamma) pathway is involved in the cilostazol effect, rats were randomized into sham, I/R, cilostazol (50 and 100 mg/kg per day, orally), pioglitazone (3 and 10 mg/kg per day, orally) and their combination at the low dose levels. Drugs regimens were administered for 14 days prior to the I/R induction. Pretreatment with cilostazol or pioglitazone provided significant protection against the I/R-induced renal injury as manifested by the attenuated serum levels of creatinine, blood urea nitrogen and cystatin C. Both drugs have also opposed the I/R-induced elevation in tissue contents/activity of neutrophil gelatinase-associated lipocalin (NGAL), kidney injury molecule-1 (Κim-1), nuclear factor-κB, interleuκin-18, caspase-1, as well as malondialdehyde, iNOS, myeloperoxidase, ICAM-1 and VCAM-1. Nevertheless, the drugs increased both the PPAR-γ transcriptional activity and the content of glutathione. Furthermore, combining the two low doses of both drugs produced effects comparable to that of the high dose level of either drug, advocating the fortification of pioglitazone renoprotective effect when given concomitantly with cilostazol. In conclusion, cilostazol purveyed conceivable novel renoprotective mechanisms and alleviated incidents associated with acute renal injury either alone or in combination with pioglitazone partially via the elevation of PPAR-γ besides the amendment of the aforementioned biomarkers.

## Introduction

Acute kidney injury (AΚI) induced by ischemia/reperfusion (I/R) is a common consequence of kidney transplantation, with high morbidity and mortality rates despite the advanced renal replacement therapy [1]. AΚI remains a critical issue because of its deleterious impacts on the graft functions, presented usually as acute rejection and diminished long-term survival [2].

The mechanism of cell damage following I/R includes an interplay between tubular and vascular abnormalities with increased release of inflammatory mediators and oxidative stress [3]. During ischemia, ATP depletion favors the loss of tubular cells polarity, apoptosis and shedding, while reperfusion is associated with the increased expression of vascular adhesion molecules and subsequent leukocytes activation and migration [4]. The recruitment of leukocytes into post-ischemic tissue is a complex event, instigated by adhesion to the endothelium and their transmigration [5]. Among the adhesion molecules that pave the way for the recruitment of leukocytes are the intracellular adhesion molecule 1 (ICAM-1), vascular cell adhesion molecule 1 (VCAM-1), P-selectin and E-selectin. These molecules are expressed by the damaged renal vascular endothelium and their counter receptor integrin, which is expressed on leukocytes [6]–[8]. The infiltration and activation of leukocytes are key hallmarks in the renal injury induced by reperfusion, possibly by two major flanks, the release of baleful cytokines and reactive oxygen species (ROS) [9].

A large number of inflammatory mediators has been detected in animal models of renal I/R; among them are the nuclear factor-kappa B (NF-κB), tumor necrosis factor-alpha (TNF-α), as well as interleukin (IL) -1, -6, and -18 [10], [11]. The expression and function of the latter has been now allied with renal I/R injury, where it enhances the production of other inflammatory mediators, such as NF-κB, besides IL-1, -4, -6, and -10 [12]. IL-18 helps also in the recruitment of inflammatory cells [13] via enhancing their expression; thus promoting leukocytes adhesion and activation [14]. The processing of pro-IL-18 into its mature form is mediated by the enzyme caspase-1; a fact that was evidenced by the protection of caspase-1 deficient mice from ischemic acute renal failure [15].

AΚI is typically diagnosed by an elevation of serum creatinine; however, there are several limitations for the use of this biomarker being affected by non-renal factors, which makes its values lag behind the degree of the renal tubular epithelial injury [16]. Over the past few years, more specific AΚI biomarkers have been identified, including serum cystatin C [17]. The latter is freely filtered by the glomerulus and is completely reabsorbed by the proximal tubule. Although it is affected by fat-free mass [18], serum cystatin C is diagnostically superior to serum creatinine and is widely accepted as a reliable biomarker for experimental renal function study [17]. Other biomarkers that are expected to play a role in tissue repair following ischemia are the neutrophil gelatinase-associated lipocalin (NGAL) [19] and the kidney injury molecule-1 (Κim-1) [20]. NGAL is up-regulated in several injury settings, including the renal tubular one and is involved in the modulation of several cellular responses, as cell proliferation, differentiation, re-epithelization and apoptosis [21], [22]. Regarding Κim-1, it is an established marker for proximal tubular damage by ischemia [23] and it acts as an adhesion molecule to reduce epithelial cells shedding [24]. Κim-1 functions as a phosphatidyl serine receptor that recognizes and binds to its ligand on the apoptotic cells and internalizes them, hence reduces tubular obstruction [25]. It also functions as a scavenger receptor, mediating the uptake of modified low density lipoprotein and necrotic cell debris [26].

Today the therapeutic effects of peroxisome proliferator activated receptor gamma (PPAR-γ) ligands, including pioglitazone, have reached far beyond their use as insulin-sensitizers, where they exert beneficial effects in conditions associated with I/R, including the kidney [27], [28]. The PPAR-γ role relies on the modulation of several I/R-connected markers, viz., apoptosis, caspase-1, NF-κB and its downstream target genes TNF-α, ICAM-1, and iNOS [28], [29]. Moreover, rosiglitazone, another PPAR-γ agonist, mediated its anti-inflammatory effect partly via inhibiting IL-18 [30].

Cilostazol, a selective phosphodiestrase3 (PDE 3) inhibitor, is currently used in the treatment of intermittent claudication [31]. It ameliorates oxidative stress and inflammation in several experimental models [32]–[35] via inhibiting the expression of ICAM-1 and VCAM-1, as well as the activation of NF-κB and the adhesion of neutrophils. Notably, *in-vitro* studies revealed that cilostazol increases the expression of PPAR-γ and its transcriptional activity in different cell types [36], [37].

To this end, the goal of the current study was to test the potential renoprotective effect of cilostazol against an I/R model, to verify the possible involvement of PPAR-γ activity, as one of its mechanistic cassette, and to study its correlation with Κim-1, NGAL, IL-18 and caspase-1. Additionally, the influence of cilostazol on the pioglitazone renoprotective action was evaluated.

## Materials and Methods

### 1. Animals

Adult male Wistar rats, weighing 200–250 gram, were used in the present study. Rats were kept under controlled conditions, with a 12 h light/dark cycles, at an ambient temperature of 22±2°C and a humidity of 65–70%. This study was carried out in strict accordance with the recommendations in the Guide for the Care and Use of Laboratory Animals of the National Institutes of Health. The study protocol was approved by the guidelines of the Research Ethical Committee of the Faculty of Pharmacy, Cairo University, Cairo, Egypt (Permit Number: PT 204). All the surgical processes were performed under xylazine/ketamine anesthesia and all efforts were made to minimize suffering.

### 2. Experimental design

Rats were randomly assigned into 7 groups (n = 6), where the first group served as the sham operated control and the second one was the I/R control group. In groups 3 and 4 the rats were pretreated with cilostazol (Otsuka Pharmaceutical Co. Ltd., Tokushima, Japan) 50 mg/kg (Cilo_50_) [38] and 100 mg/kg (Cilo_100_) [39] dose levels, respectively. The animals in groups 5 and 6 were pretreated with pioglitazone (Eli Lilly and Company, Indiana, USA) 3 mg/kg (Pio_3_) [40] and 10 mg/kg (Pio_10_) [41], respectively. Finally, animals of the last group were administered a combination of the low doses of pioglitazone and cilostazol. All treatment regimens were administered p.o. for two weeks and the I/R was induced on day 15.

### 3. Induction of renal I/R injury

Renal I/R was induced in rats as previously described [42]. Briefly, rats were anaesthetized with xylazine (10 mg/kg) and ketamine (75 mg/kg). After a midline abdominal incision both renal pedicles were isolated and bilateral renal ischemia was induced by placing a microvascular clamp over each pedicle for 45 min. At the end of the ischemic period the clamps were removed and the abdominal incision was sutured and the rats were kept to recover from anesthesia. The animals were kept well hydrated with saline and the body temperature was kept at 37°C by placing the animals over a heating pad. The sham-operated group underwent the same surgical conditions without performing I/R. At the end of the 24 hours reperfusion, animals were re-anesthetized and blood was collected from the vena cava. Thereafter, both kidneys were quickly removed and stored at −80°C until the analysis of the biochemical parameters.

### 4. Renal function study

Blood samples were centrifuged to separate clear sera, which were used for the colorimetric assessment of creatinine and blood urea nitrogen (BUN). Serum creatinine was quantified by a standard method based on a modified Jaffé technique [43] using Olympus AU400 automated analyzer (Olympus, Tokyo, Japan). Simply, clear sera were processed with working reagent of sodium hydroxide (120 mmol/l) and picric acid (9.2 mmol/L) and the absorbance of the developed color was measured automatically at precise time interval. BUN was manually assessed using the purchased kit (Stanbio, Texas, USA), which depends on the enzymatic Berthelot reaction for the BUN estimation [44]. Serum cystatin C level was determined by enzyme-linked immunosorbent assay technique using rat ELISA kit (Immunology Consultant Laboratory, Oregon, USA) and processed according to the manufacturer instructions.

### 5. Renal tissue preparation

For each animal, the right kidney was weighed and homogenized in ice-cold saline (10% w/v) and the homogenate was then divided into several aliquots for the assessment of redox/nitrosative parameters, inflammatory and immunomodulatory markers, as well as Κim-1 and NGAL. The left kidney was used for the preparation of nuclear extract for the assessment of PPAR-γ transcriptional activity.

#### 5.1. Assessment of renal Kim-1 and NGAL

Renal Kim-1 and NGAL were measured in tissue homogenate using specific ELISA kits (USCN Life Science Inc., Wuhan, China) according to the manufacturer's prescripts.

#### 5.2. Assessment of renal glutathione (GSH) and lipid peroxidation

Renal GSH was determined according to Ellman's method as previously described [45]. After precipitating the SH-containing proteins, the yellow color developed by the reduced Ellman's reagent was measured colorimetrically at 412 nm. The method described by Mihara and Uchiyama [46] was adopted for the assessment of the lipid peroxidation product, malondialdehyde (MDA). This is based on the reaction of tissue MDA with thiobarbituric acid to produce a pink pigment that was extracted by n-butanol and measured colorimetrically at 520 and 535 nm.

#### 5.3. Assessment of renal inducible nitric oxide synthase (iNOS)

According to the method described by the manufacturer, the tissue iNOS was assayed using the purchased ELISA assay kit (Bioassay Technology Laboratory, Shanghai, China).

#### 5.4. Estimation of renal myeloperoxidase (MPO) activity, ICAM-1 and VCAM-1

Tissue MPO activity is used for the assessment of neutrophil infiltration. As described by Bradley et al. [47], renal MPO was extracted by suspending the tissue homogenate after centrifugation at 14,000 rpm for 15 minutes at 4°C in 1.25 ml of phosphate-HTAB buffer (pH 6), followed by three freeze/thaw cycles. The measurement of the MPO activity is based on its ability to catalyze the oxidation of o-dianisidine at pH 6 by hydrogen peroxide, resulting in the formation of colored compound that exhibits an increased absorbance at 460 nm. The renal contents of ICAM-1 and VCAM-1 were quantified by ELISA technique, using the purchased kits (Wuhan Eiaab Science Co., Wuhan, China and Kamiya Biomedical Co., Washington, U.S.A, respectively).

#### 5.5. Assessment of renal NF-κB, IL-18, and caspase-1

NF-κB, IL-18, and caspase-1 were estimated according to the manufacturer's instructions provided by the ELISA assay kits (USCN Life Science Inc., Wuhan, China).

#### 5.6. PPAR-γ Transcriptional Activity

The transcriptional activity of PPAR-γ was assessed using the purchased PPAR-γ transcription factor assay kit (Abcam, Cambridge, USA). After preparation of the nuclear extract from the left kidneys, according to the method described by the manufacturer and using the nuclear extraction kit (Abcam, Cambridge, USA), the nuclear extract was added to the provided wells coated with specific oligonucleotide sequences. A primary polyclonal anti-PPAR-γ antibody was then added, followed by the addition of horseradish peroxidase-conjugated antibody and the 3,3′,5,5′ -tetramethylbenzidine substrate. The absorbance of the developed color was read at 450 nm using a microplate reader.

### 6. Statistical Analysis

Data are reported as means ± S.E.M (n = 6). Statistical differences were executed using one-way analysis of variance (ANOVA), followed by Tukey multiple comparison test. The differences were considered to be significant at *P*<0.05.

## Results

### 1. Kidney function biomarkers

**Figure 1:**
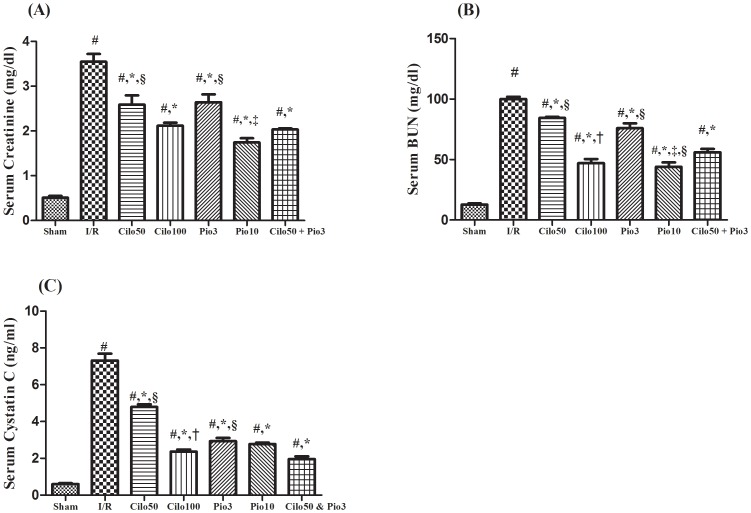
Effect of cilostazol (50 & 100 mg/kg; Cilo_50_ & Cilo_100_), pioglitazone (3 & 10 mg/kg; Pio_3_ & Pio_10_), and their combination (Cilo_50_ & Pio_3_) on the serum (A) Creatinine, (B) BUN, and (C) Cystatin C in rats subjected to ischemia (45 min)/reperfusion (24 hrs). Drugs were administered orally for 14 days then subjected to ischemia/reperfusion. Values are expressed as mean ± S.E.M (n = 6). Data are compared with sham operated control (#), I/R control (*), Cilo_50_ (†), Pio_3_ (‡), and combination (§) pretreated groups (one-way ANOVA followed by Tukey Multiple Comparison Test) at P<0.05.

**Figure 2:**
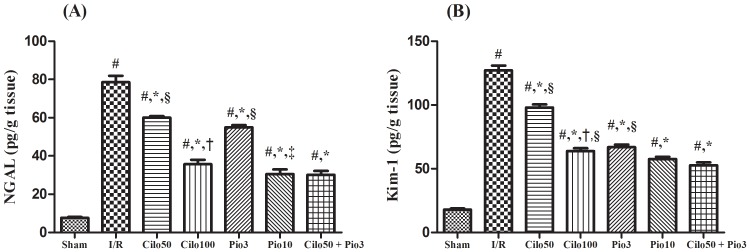
Effect of cilostazol (50 & 100 mg/kg; Cilo_50_ & Cilo_100_), pioglitazone (3 & 10 mg/kg; Pio_3_ & Pio_10_), and their combination (Cilo_50_ & Pio_3_) on renal content of (A) NGAL, and (B) Kim-1 in rats subjected to ischemia (45 min)/reperfusion (24 hrs). Drugs were administered orally for 14 days then subjected to ischemia/reperfusion. Values are expressed as mean ± S.E.M (n = 6). Data are compared with sham operated control (#), I/R control (*), Cilo_50_ (†), Pio_3_ (‡), and combination (§) pretreated groups (one-way ANOVA followed by Tukey Multiple Comparison Test) at P<0.05.

The drugs studied in the current work showed no significant effect on the kidney function biomarkers when tested in normal healthy rats (Fig. S1 A–C and Fig. S2 A & B). Hence, the effect of the treatment regimes was compared to the sham operated control group. As presented in figure 1 the I/R-induced renal injury aggravated the serum levels of creatinine (A), BUN (B) and cystatin C (C) by 13.8, 7.8, and 12 folds, respectively as compared to the sham operated control group. However, these effects were significantly suppressed by the pretreatment with cilostazol or pioglitazone in a dose dependent manner. Combining both drugs in the small dose level mediated an effect comparable to that shown by the high dose for each drug alone. The same pattern was also observed in the renal content of other specific kidney injury markers, viz., NGAL and Kim-1, as depicted in figure 2A & B. The level of these parameters was magnified in the injured non-treated kidney by 10.3 and 7.01 folds, respectively and as compared to the sham group. Nevertheless, cilostazol and pioglitazone have opposed the I/R effect significantly, in a dose related order. The combined low doses mediated an effect similar to that shown by Pio_10_ on the two assessed parameters.

### 2. Effect on renal oxidative and nitosative stress parameters

**Figure 3:**
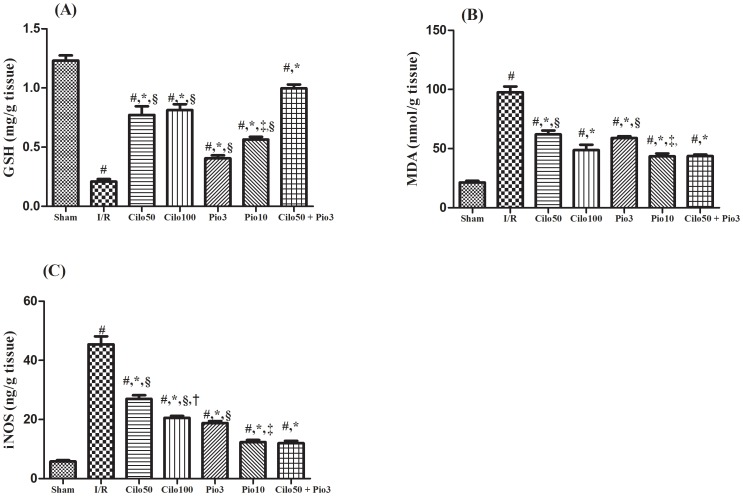
Effect of cilostazol (50 & 100 mg/kg; Cilo_50_ & Cilo_100_), pioglitazone (3 & 10 mg/kg; Pio_3_ & Pio_10_), and their combination (Cilo_50_ & Pio_3_) on the renal content of (A) GSH, (B) MDA and (C) iNOSin rats subjected to ischemia (45 min)/reperfusion (24 hrs). Drugs were administered orally for 14 days then subjected to ischemia/reperfusion. Values are expressed as mean ± S.E.M (n = 6). Data are compared with sham operated control (#), I/R control (*), Cilo_50_ (†), Pio_3_ (‡), and combination (§) pretreated groups (one-way ANOVA followed by Tukey Multiple Comparison Test) at P<0.05.

The I/R insult has impoverished the renal GSH content, while boosted that of MDA (4.5 folds), as compared to the sham group, effects that were reverted by the two tested drugs (Fig. 3A & B). The combination regimen showed the most superior effect in reviving renal GSH contents among the pre-treated groups, where I/R reduced its level by 23% only as compared to the sham operated group. Figure 3C depicted the marked elevation in the iNOS (8 folds) in the untreated I/R injured rats; however, the two drugs used halted the I/R effect in a dose dependent manner and their combination regimen offered an effect similar to that mediated by Pio_10_. On the other hand, figure S3A–C showed that the tested drugs had no effect on the renal oxidative and nitosative stress parameters when pre-administered in normal healthy rats.

### 3. Effect on leukocytes infiltration markers

**Figure 4:**
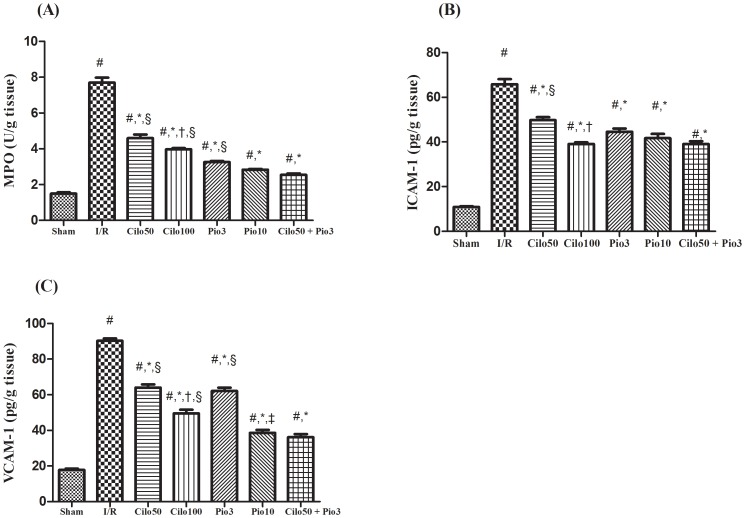
Effect of cilostazol (50 & 100 mg/kg; Cilo_50_ & Cilo_100_), pioglitazone (3 & 10 mg/kg; Pio_3_ & Pio_10_), and their combination (Cilo_50_ & Pio_3_) on the renal (A) MPO activity, and renal contents of (B) ICAM-1 and (C) VCAM-1in rats subjected to ischemia (45 min)/reperfusion (24 hrs). Drugs were administered orally for 14 days then subjected to ischemia/reperfusion. Values are expressed as mean ± S.E.M (n = 6). Data are compared with sham operated control (#), I/R control (*), Cilo_50_ (†), Pio_3_ (‡), and combination (§) pretreated groups (one-way ANOVA followed by Tukey Multiple Comparison Test) at P<0.05.

After the induction of the I/R insult the contents/activity of MPO, ICAM-1, and VCAM-1 were markedly elevated by 5–6 folds. Pretreatment with Cilo_100_ showed a better effect than Cilo_50_ on the 3 assessed parameters; however, the opposing dose dependent effect of pioglitazone was noticed only on the VCAM-1 content. The combination regimen offered an effect similar to that mediated by Pio_10_ regarding the 3 parameters (Fig. 4A, B & C). On the contrary, neither the tested drugs nor their combination altered the leukocytes infiltration markers when given to normal healthy rats, as compared to sham operated animals (Fig. S4A, B & C).

### 4. Effect on inflammatory mediators

**Figure 5:**
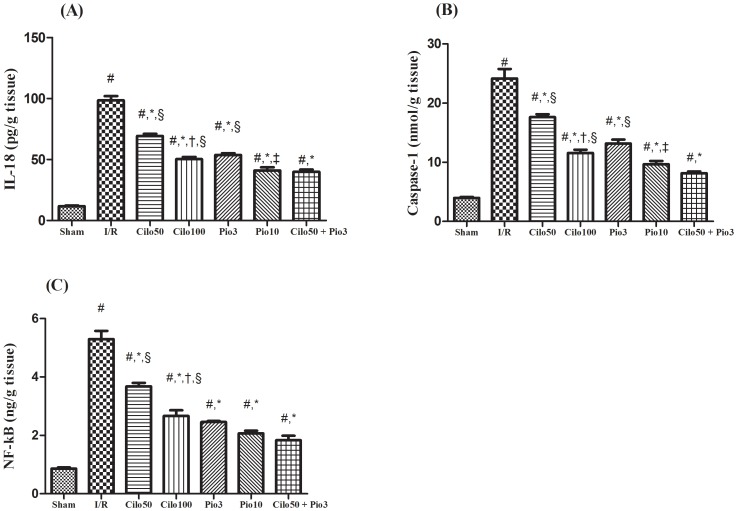
Effect of cilostazol (50 & 100 mg/kg; Cilo_50_& Cilo_100_), pioglitazone (3 & 10 mg/kg; Pio_3_ & Pio_10_), and their combination (Cilo_50_& Pio_3_) on the renal content of (A) IL-18, (B) caspase-1 and (C) NF-κBin rats subjected to ischemia (45 min)/reperfusion (24 hrs). Drugs were administered orally for 14 days then subjected to ischemia/reperfusion. Values are expressed as mean ± S.E.M (n = 6). Data are compared with sham operated control (#), I/R control (*), Cilo_50_ (†), Pio_3_ (‡), and combination (§) pretreated groups (one-way ANOVA followed by Tukey Multiple Comparison Test) at P<0.05.

Figure 5 (A, B & C) illustrates the boosting effect of the I/R injury on the contents of IL-18 (8 folds), caspase-1 (6 folds) and NF-κB (6 folds) in the renal tissues after 24 hour reperfusion. The I/R effect was significantly hindered in the treated groups according to the dose level tested, except for the effect of the 2 dose levels of pioglitazone on the NF-κB content. Again the combination regimen showed an effect equal to that mediated by the high dose of pioglitazone. As depicted in figure S5 and its panels (A, B & C) the aforesaid inflammatory mediators were not altered when the two tested drugs or their combination were administered to normal animals.

### 5. Effect on PPAR-γ activity

**Figure 6:**
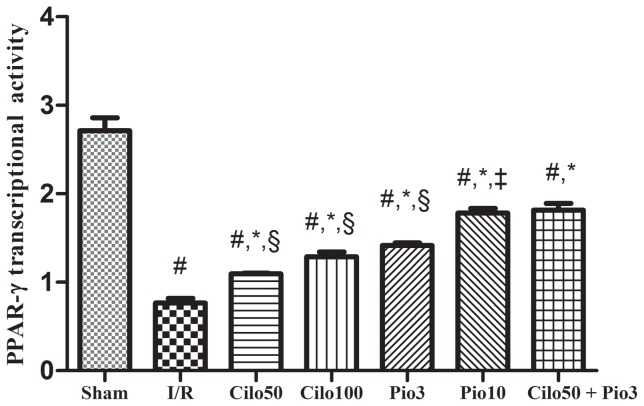
Effect of cilostazol (50 & 100 mg/kg; Cilo_50_& Cilo_100_), pioglitazone (3 & 10 mg/kg; Pio_3_ & Pio_10_), and their combination (Cilo_50_& Pio_3_) on the PPAR-*γ* transcription activity in rats subjected to ischemia (45 min)/reperfusion (24 hrs). Drugs were administered orally for 14 days then subjected to ischemia/reperfusion. Values are expressed as mean ± S.E.M (n = 6). Data are compared with sham operated control (#), I/R control (*), Cilo_50_ (†), Pio_3_ (‡), and combination (§) pretreated groups (one-way ANOVA followed by Tukey Multiple Comparison Test) at P<0.05.

In the sham-operated group there is a normal presence of PPAR-γ in robust renal tissue, which was not affected by any of the pretreatment regimen (Fig. S6). Conversely, the 24 hours reperfusion diminished the level of PPAR-γ by 71.6%, while the pretreatment with cilostazol, at the two dose levels, increased PPAR-γ by 42.8 and 67.5%, respectively. The pioglitazone effect, however, overrides that of cilostazol and elevated PPAR-γ by 83.1 and 131%, respectively; the latter effect was also attained by the addition of Cilo_50_ to Pio_3_, as presented in figure 6.

### 6. Correlation among parameters

In this work a strong correlation has been detected between PPAR-γ, NGAL, and Kim-1, with PPAR-γ, iNOS, IL-18 and caspase-1, as detailed in table 1.

**Table 1:** Analysis of the correlation coefficient (*r*) between PPAR-γ, NGAL, andKIM-1, with PPAR-γ, iNOS, IL-18 and caspase-1.

	PPAR-γ	i-NOS	IL-18	Caspase-1
PPAR-γ	–	0.95	0.94	0.95
		P = 0.002	P = 0.003	P = 0.002
NGAL	−0.89	0.96	0.98	0.98
	P = 0.009	P = 0.001	P<0.001	P<0.001
KIM-1	−0.93	0.97	0.99	0.99
	P = 0.004	P = 0.001	P<0.001	P<0.001

Correlation was carried out in untreated I/R and pre-treated I/R animals. Table correlation was performed using Pearson's correlation equation.

## Discussion

The renal I/R injury is a grave clinical problem, which despite the development in critical care medicine, is still associated with momentous morbidity and mortality [1]. The I/R insult is considered as a biphasic mystery in which the ischemic episode turns out ATP depletion and the consequent blood reperfusion aggravates the tissue injury [48]. The renal I/R-induced glomerular injury has been linked to the I/R-mediated podocytes effacement, cytoplasmic oedema, detachment, and even cell death [49]. These events may explain the elevated serum level of the BUN, creatinine and cystatin C in the I/R untreated group. To our knowledge, the current study is the first to address the possible renoprotective mechanisms interceded by cilostazol in renal I/R rat model. Cilostazol pretreatment improved renal glomerular filtration function as confirmed by the inhibition of the serum levels of creatinine, BUN, and cystatin C. The same results have been seen in the pioglitazone pretreated groups, effects that coincide with the recent findings of Zou et al. [50]. The ability of pioglitazone to prevent podocytes apoptosis may be partially behind its positive effect [49].

The renoprotective effect of cilostazol and pioglitazone was further confirmed, for the first time, using more specific injury markers viz., Κim-1and NGAL. Κim-1 is expressed in low levels in the normal kidney and is highly upregulated in the proximal tubules after ischemic AΚI [23]. This fact supports our finding, which upholds also with previous studies in different kidney injury models [23], [51]. The tissue level of NGAL was also discernibly increased following the I/R insult, mimicking previous studies [52], [53] and implying that NGAL is derived from the damaged nephrons [54]. The increased amount of Κim-1 and NGAL may possibly act as a compensatory mechanism, where the former is responsible for the tissue re-epithelization and reduction of apoptosis [21], [22], while the latter acts as an adhesion molecule to reduce the epithelial shedding [24]. Cilostazol, pioglitazone, and their combination regimen leveled off both markers, indicating, thus, the reduced intensity of the renal injury, the decrease of damaged nephrons, and the improved renal function.

The injurious events that accompany the I/R insult include an increase in the oxidative/nitrosative stress biomarkers, recruitment of leukocytes, and the release of redundant levels of pro-inflammatory mediators [3], [4]. The provenance of ROS is believed to be the xanthine oxidase system [55] and the infiltrated leukocytes [4]. The ROS harmful effect is mediated by the peroxidation of the cell membrane lipids and oxidative damage of proteins, as well as DNA, leading finally to cell death [56].

Cilostazol opposed the I/R-induced redox tissue imbalance, where it reduced the elevated level of MDA and preserved that of GSH, possibly via its free radical scavenging property and/or by increasing the activity of the endogenous antioxidants. These findings are in agreement with earlier studies on lung injury induced by lower limb I/R [57] and spinal cord I/R-induced injury [58]. These reports support the possible antioxidant capacity of cilostazol, which could be one of its renoprotective mechanistic pathways. Moreover, the current results verified the pioglitazone ability to reduce oxidative stress and its deleterious leverage on renal tissue, where it reduced the tissue level of MDA and enhanced that of GSH. Our data mimicked that reported previously by Shah et al. [59] and Zou et al. [50] in an experimental model of renal I/R, which attested the antioxidant activity of pioglitazone in diminishing renal injury. Nevertheless, combining both drugs, at the low dose level, boosted the GSH content significantly even above the effect mediated by the high doses, indicating a possible synergistic effect between the two drugs.

Nitric oxide (NO) is an important signaling molecule that is produced by the nitric oxide synthase (NOS) enzymes isoforms [60]. During the renal I/R, the inducible isoform, iNOS, is highly induced, leading to the excessive generation of NO, which activates oxygen-radical-mediated injury via generating the peroxynitrite anion [61]. In the present study, we raveled out that cilostazol and pioglitazone alone or in combination can cause significant reduction in the nitrosative stress via decreasing the tissue level of iNOS, suggesting another tool for the cilostazol renoprotective effect.

There is ample evidence that the infiltration of neutrophils, among other leukocytes, mediates tubular injury and plays a key role in the development of acute renal failure (ARF) [62], where depletion of the peripheral neutrophils mediates protection against ARF and the use of an anti-ICAM-1 therapy offered a beneficial role in ARF [6]. These facts are in line with our findings in which I/R insult provoked neutrophils invasion. Cilostazol pretreatment reduced MPO activity, which is a telltale for neutrophils invasion and inflammation, as well as the adhesion molecules ICAM-1 and VCAM-1; these results corroborated with another study on the focal cerebral I/R injury [63]. In support to our findings, cilostazol effectively reduced the expression of ICAM-1 and was able to suppress the leucocyte-endothelial cell interactions in an experimental model of retinal ischemia [33].

Besides its anti-inflammatory effect, cilostazol possesses immunomodulatory properties, where it was able to lessen the I/R-induced elevation of NF-κB, which targets myriad of chemokines, cytokines, and adhesion molecules. Renal I/R is typically associated with marked release of motley inflammatory mediators, among which is IL-18 [11] that induces the expression of both NF-κB and ICAM-1[12], [14]. Hence, cilostazol by inhibiting IL-18, as documented in this work, can explain the reduction in NF-κB and the neutrophils adhesion and invasion. Previous study defends our findings, in which cilostazol inhibited the activation of NF-κB in the aorta isolated from diabetic rats, indicating the possible anti-inflammatory/immunomodulatory effect of cilostazol via suppressing the production of NF-κB [64].

Our study revealed also that pioglitazone, similarly, suppresses inflammatory events associated with renal I/R; results that are compatible with previous studies that investigate the renoprotective activity attained by pioglitazone in experimental models of renal I/R [30]. Pioglitazone, at both dose levels, reduced the activity/content of MPO, VCAM-1 and ICAM-1 in renal tissue experiencing reperfusion injury. The two doses suppressed also the level of IL-18 and NF-κB, thereby defeat the I/R induced release of pro-inflammatory cytokines and mitigate the attendant inflammation. To the authors' knowledge, this is the first study to assess the effect of pioglitazone on these biomarkers in a rat model of renal I/R.

The caspase-1 enzyme, which is responsible for the activation of the pro-inflammatory cytokines, viz., IL-1 and 18, was markedly elevated in the current I/R group [65], a finding that can explain the I/R-induced IL-18 elevation. However, pretreatment with either drug inhibited its level in a dose dependent pattern, offering another validation for the renoprotective effect of both drugs. Inhibition or deficiency of caspase-1 was reported to protect kidney function and histology against ischemic ARF and this protection was associated with a decreased conversion of IL-18 precursor to the mature form in the kidney [65]. As caspase-1-deficiency accompanies also decreased neutrophil infiltration [65], thus, it is suggested that the deleterious role of IL-18 may be due, in part, to the increased neutrophil infiltration.

With reference to MPO and IL-18, combination of the low doses of cilostazol and pioglitazone lowered these inflammatory biomarkers to a sphere better than that shown by each drug alone or even Cilo_100_. Regarding NF-κB, the combination regimen lowered its content more than that attained by Cilo_100_; however, there was no upshot achieved by this combination in comparison with pioglitazone alone at its two dose levels.

Furthermore, the PPAR-γ transcriptional activity was reduced with a 24 hours reperfusion, a finding that is in agreement with that of Yoshimura and his colleagues [66], who stated that PPAR-γ expression is reduced with longer periods of reperfusion, up to 24 hours. The present study also has linked the renoprotective effect of cilostazol, at its two dose levels, to the enhancement of the PPAR-γ transcription activity. In addition, we showed that combining the two drugs at the low dose levels mimicked the effect of Pio_10_ in stimulating the PPAR-γ transcriptional activity; such an effect points to a potential synergistic effect between the two drugs.

As previous studies have connected the changes in the previously measured parameters with the activation of the PPAR-γ in different organs [28], [30], [67], the current study has verified the strong correlation between the aforementioned parameters and the activity of PPAR-γ transcription in renal tissues, hence, supporting the possible dependence or link of these biomarkers to PPAR-γ.

In summary, our results revealed that I/R-induced renal injury can be protected by cilostazol, via modulating the I/R-effect on oxidative stress, iNOS, NF-κB, IL-18, caspase-1, NGAL, Κim-1and PPAR-γ in renal tissues. Moreover, the combination of the low doses of cilostazol with pioglitazone showed a better renoprotective effect that highlighted the positive role of this combination.

## Supporting Information

Figure S1 Effect of cilostazol (50 & 100 mg/kg; Cilo_50_ & Cilo_100_), pioglitazone (3 & 10 mg/kg; Pio_3_ & Pio_10_), and their combination (Cilo_50_ & Pio_3_) on the serum (A) Creatinine, (B) BUN, and (C) Cystatin C in sham operated rats. Drugs were administered orally for 14 days then subjected to sham operation. Values are expressed as mean ± S.E.M (n = 6) and analyzed using one-way ANOVA followed by Tukey Multiple Comparison Test, P<0.05.
(TIF)

Figure S2 Effect of cilostazol (50 & 100 mg/kg; Cilo_50_ & Cilo_100_), pioglitazone (3 & 10 mg/kg; Pio_3_ & Pio_10_), and their combination (Cilo_50_ & Pio_3_) on renal content of (A) NGAL, and (B) Kim-1 in sham operated rats. Drugs were administered orally for 14 days then subjected to sham operation. Values are expressed as mean ± S.E.M (n = 6) and analyzed using one-way ANOVA followed by Tukey Multiple Comparison Test, P<0.05.
(TIF)

Figure S3 Effect of cilostazol (50 & 100 mg/kg; Cilo_50_ & Cilo_100_), pioglitazone (3 & 10 mg/kg; Pio_3_ & Pio_10_), and their combination (Cilo_50_ & Pio_3_) on the renal content of (A) GSH, (B) MDA and (C) iNOS in sham operated rats. Drugs were administered orally for 14 days then subjected to sham operation. Values are expressed as mean ± S.E.M (n = 6) and analyzed using one-way ANOVA followed by Tukey Multiple Comparison Test, P<0.05.
(TIF)

Figure S4 Effect of cilostazol (50 & 100 mg/kg; Cilo_50_ & Cilo_100_), pioglitazone (3 & 10 mg/kg; Pio_3_ & Pio_10_), and their combination (Cilo_50_ & Pio_3_) on the renal (A) MPO activity, and renal contents of (B) ICAM-1 and (C) VCAM- in sham operated rats. Drugs were administered orally for 14 days then subjected to sham operation. Values are expressed as mean ± S.E.M (n = 6) and analyzed using one-way ANOVA followed by Tukey Multiple Comparison Test, P<0.05.
(TIF)

Figure S5 Effect of cilostazol (50 & 100 mg/kg; Cilo_50_& Cilo_100_), pioglitazone (3 & 10 mg/kg; Pio_3_ & Pio_10_), and their combination (Cilo_50_& Pio_3_) on the renal content of (A) IL-18, (B) caspase-1 and (C) NF-κB in sham operated rats. Drugs were administered orally for 14 days then subjected to sham operation. Values are expressed as mean ± S.E.M (n = 6) and analyzed using one-way ANOVA followed by Tukey Multiple Comparison Test, P<0.05.
(TIF)

Figure S6 Effect of cilostazol (50 & 100 mg/kg; Cilo_50_& Cilo_100_), pioglitazone (3 & 10 mg/kg; Pio_3_ & Pio_10_), and their combination (Cilo_50_& Pio_3_) on the PPAR-*γ* transcription activity in sham operated rats. Drugs were administered orally for 14 days then subjected to sham operation. Values are expressed as mean ± S.E.M (n = 6) and analyzed using one-way ANOVA followed by Tukey Multiple Comparison Test, P<0.05. The authors ensure that the production can use this reference to link the reader to the Supporting Information layouts.
(TIF)
