# Supplementary figures and images for: Cilostazol Renoprotective Effect: Modulation of PPAR-γ, NGAL, KIM-1 and IL-18 Underlies Its Novel Effect in a Model of Ischemia-Reperfusion
Source: PLoS One. 2014 May 9;9(5):e95313. doi: 10.1371/journal.pone.0095313 (PMC4015937; doi:10.1371/journal.pone.0095313)

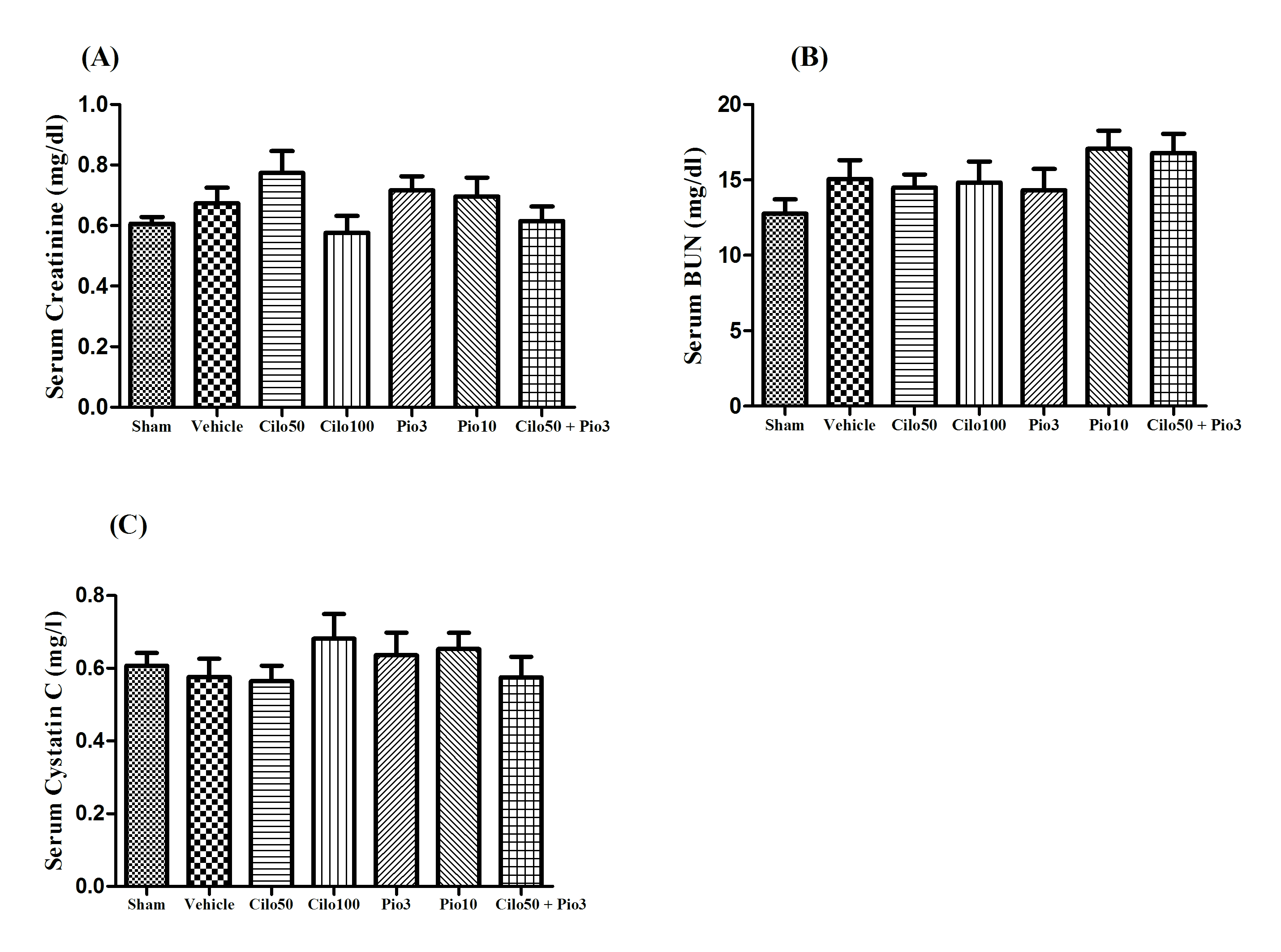

Supplement: Figure S1 — Effect of cilostazol (50 & 100 mg/kg; Cilo50 & Cilo100), pioglitazone (3 & 10 mg/kg; Pio3 & Pio10), and their combination (Cilo50 & Pio3) on the serum (A) Creatinine, (B) BUN, and (C) Cystatin C in sham operated rats. Drugs were administered orally for 14 days then subjected to sham operation. Values are expressed as mean ± S.E.M (n = 6) and analyzed using one-way ANOVA followed by Tukey Multiple Comparison Test, P<0.05. (TIF) [file pone.0095313.s001.tif]

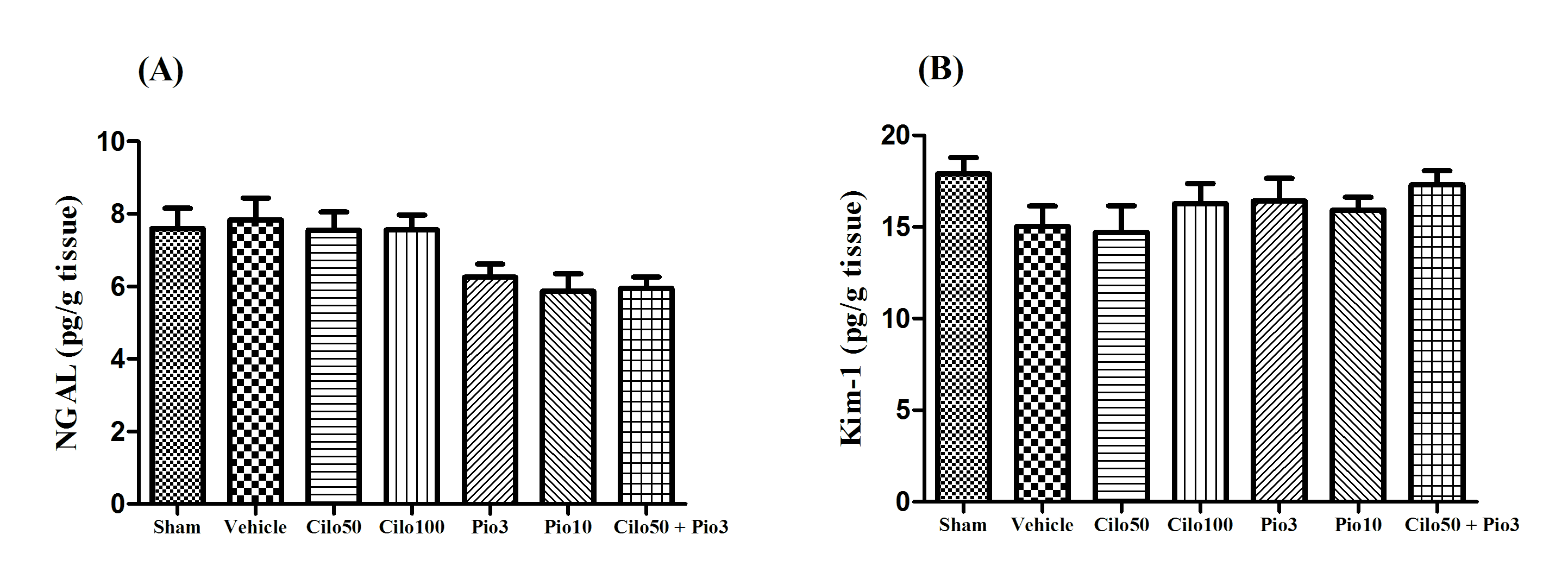

Supplement: Figure S2 — Effect of cilostazol (50 & 100 mg/kg; Cilo50 & Cilo100), pioglitazone (3 & 10 mg/kg; Pio3 & Pio10), and their combination (Cilo50 & Pio3) on renal content of (A) NGAL, and (B) Kim-1 in sham operated rats. Drugs were administered orally for 14 days then subjected to sham operation. Values are expressed as mean ± S.E.M (n = 6) and analyzed using one-way ANOVA followed by Tukey Multiple Comparison Test, P<0.05. (TIF) [file pone.0095313.s002.tif]

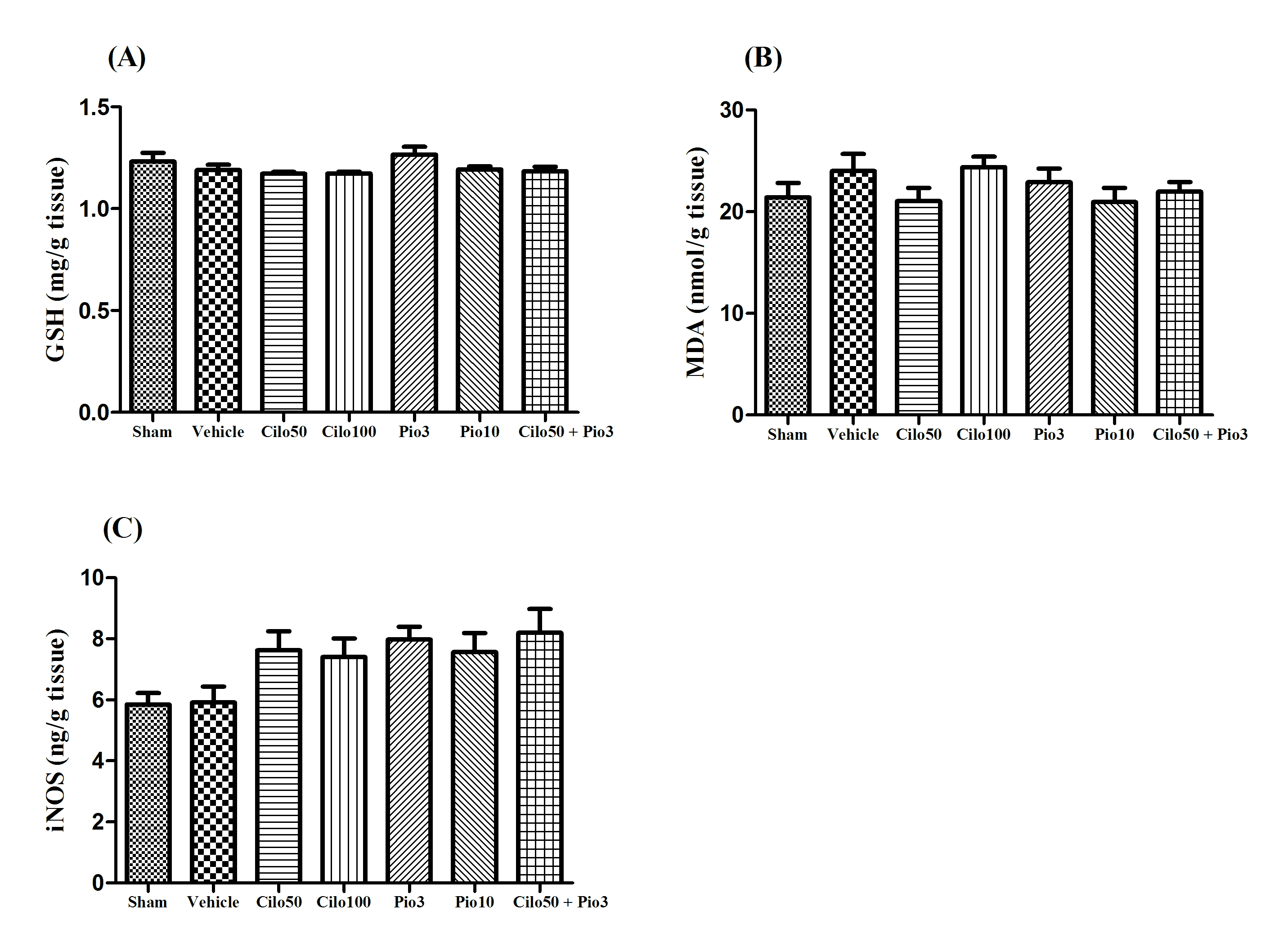

Supplement: Figure S3 — Effect of cilostazol (50 & 100 mg/kg; Cilo50 & Cilo100), pioglitazone (3 & 10 mg/kg; Pio3 & Pio10), and their combination (Cilo50 & Pio3) on the renal content of (A) GSH, (B) MDA and (C) iNOS in sham operated rats. Drugs were administered orally for 14 days then subjected to sham operation. Values are expressed as mean ± S.E.M (n = 6) and analyzed using one-way ANOVA followed by Tukey Multiple Comparison Test, P<0.05. (TIF) [file pone.0095313.s003.tif]

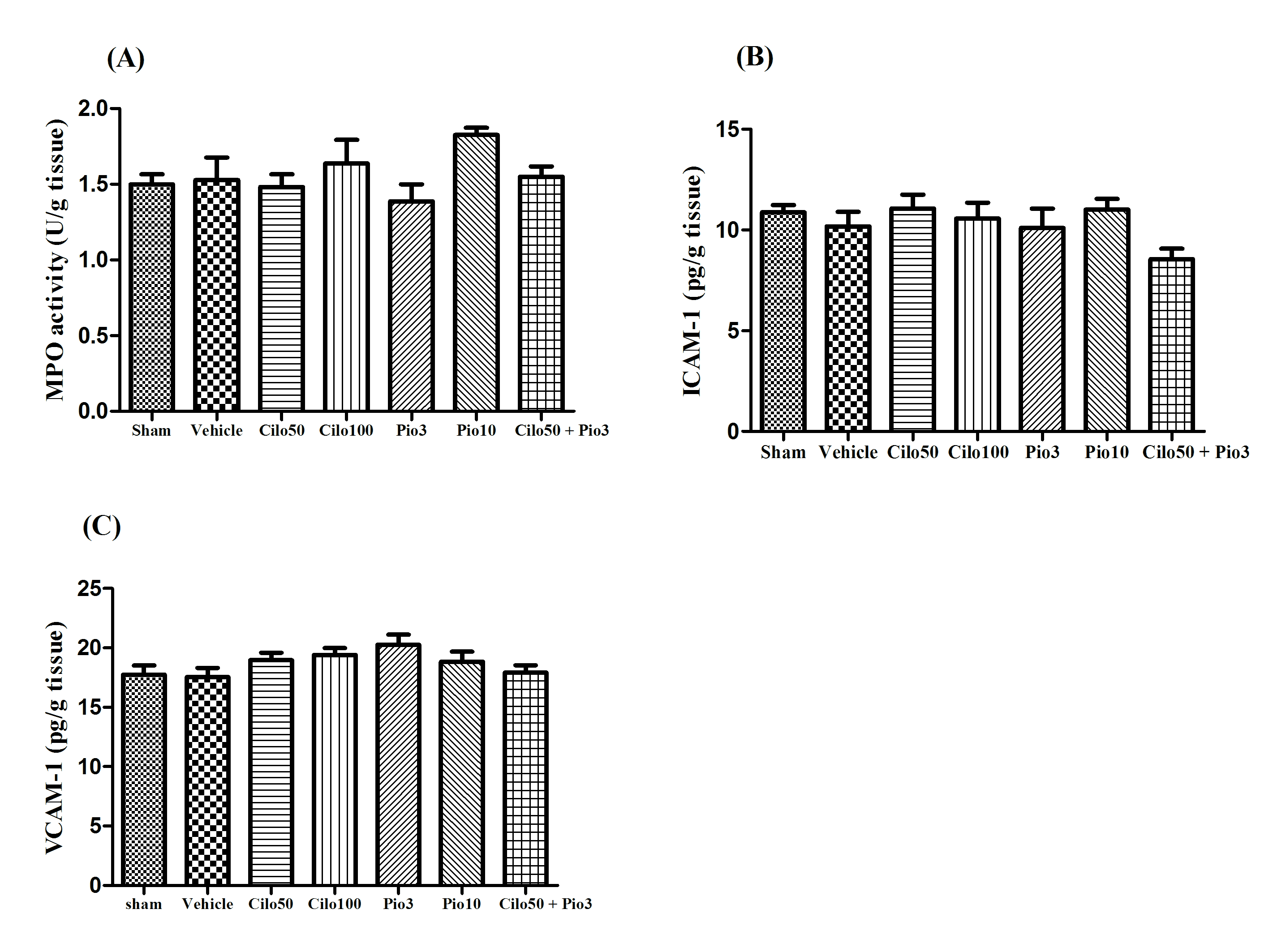

Supplement: Figure S4 — Effect of cilostazol (50 & 100 mg/kg; Cilo50 & Cilo100), pioglitazone (3 & 10 mg/kg; Pio3 & Pio10), and their combination (Cilo50 & Pio3) on the renal (A) MPO activity, and renal contents of (B) ICAM-1 and (C) VCAM- in sham operated rats. Drugs were administered orally for 14 days then subjected to sham operation. Values are expressed as mean ± S.E.M (n = 6) and analyzed using one-way ANOVA followed by Tukey Multiple Comparison Test, P<0.05. (TIF) [file pone.0095313.s004.tif]

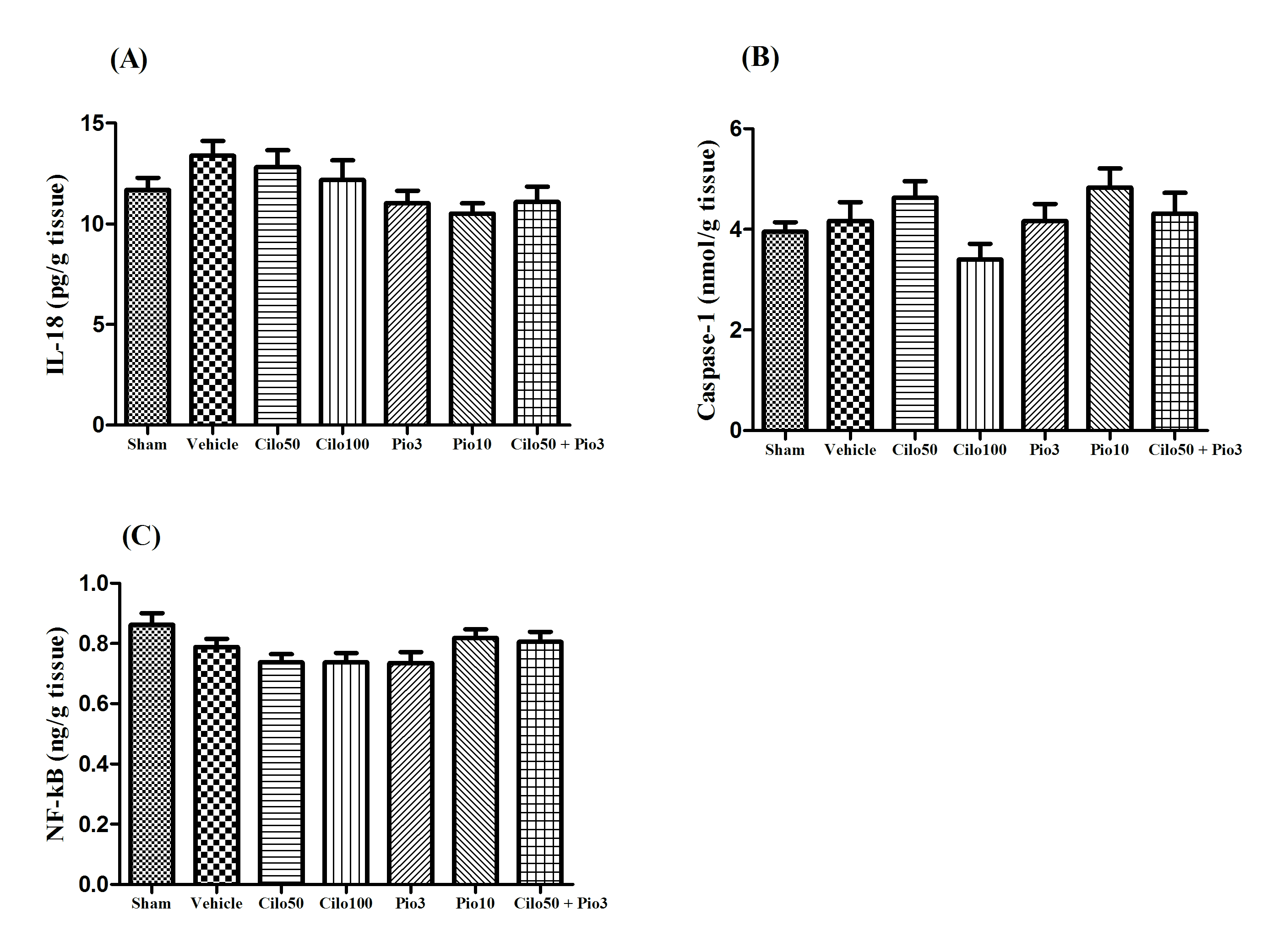

Supplement: Figure S5 — Effect of cilostazol (50 & 100 mg/kg; Cilo50& Cilo100), pioglitazone (3 & 10 mg/kg; Pio3 & Pio10), and their combination (Cilo50& Pio3) on the renal content of (A) IL-18, (B) caspase-1 and (C) NF-κB in sham operated rats. Drugs were administered orally for 14 days then subjected to sham operation. Values are expressed as mean ± S.E.M (n = 6) and analyzed using one-way ANOVA followed by Tukey Multiple Comparison Test, P<0.05. (TIF) [file pone.0095313.s005.tif]

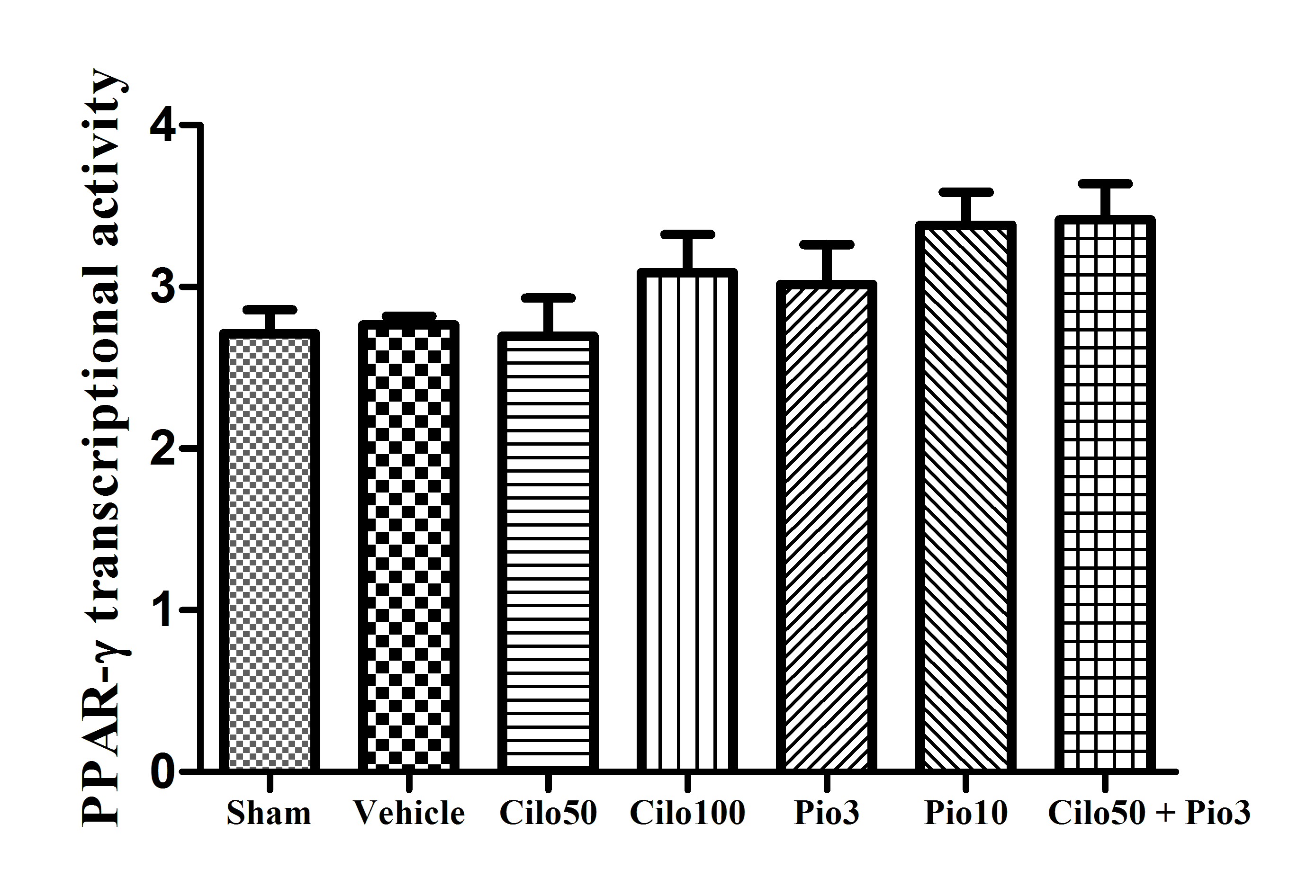

Supplement: Figure S6 — Effect of cilostazol (50 & 100 mg/kg; Cilo50& Cilo100), pioglitazone (3 & 10 mg/kg; Pio3 & Pio10), and their combination (Cilo50& Pio3) on the PPAR-γ transcription activity in sham operated rats. Drugs were administered orally for 14 days then subjected to sham operation. Values are expressed as mean ± S.E.M (n = 6) and analyzed using one-way ANOVA followed by Tukey Multiple Comparison Test, P<0.05. The authors ensure that the production can use this reference to link the reader to the Supporting Information layouts. (TIF) [file pone.0095313.s006.tif]
